# Screening for Best Neuronal-Glial Differentiation Protocols of Neuralizing Agents Using a Multi-Sized Microfluidic Embryoid Body Array

**DOI:** 10.3390/pharmaceutics14020339

**Published:** 2022-01-31

**Authors:** Christoph Eilenberger, Mario Rothbauer, Konstanze Brandauer, Sarah Spitz, Eva-Kathrin Ehmoser, Seta Küpcü, Peter Ertl

**Affiliations:** 1 Faculty of Technical Chemistry, Vienna University of Technology, Getreidemarkt 9, 1060 Vienna, Austria; konstanze.brandauer@tuwien.ac.at (K.B.); sarah.spitz@tuwien.ac.at (S.S.); peter.ertl@tuwien.ac.at (P.E.); 2 Orthopedic Microsystems, Karl Chiari Lab for Orthopaedic Biology, Department of Orthopedics and Trauma Surgery, Medical University of Vienna, Währinger Gürtel 18-20, 1090 Vienna, Austria; 3 Department of Nanobiotechnology, Institute of Synthetic Bioarchitectures, University of Natural Resources and Life Sciences, Muthgasse 18, 1190 Vienna, Austria; eva.ehmoser@boku.ac.at (E.-K.E.); seta.kuepcue@boku.ac.at (S.K.)

**Keywords:** embryoid body, microfluidics, organ-on-a-chip, neural differentiation, EC23, retinoic acid

## Abstract

Stem cell technology and embryonic stem cell models are of great interest in biomedical research since they provide deeper insights into, e.g., neurogenesis and early mammalian brain development. Despite their great scientific potential, the reliable establishment of three-dimensional embryoid bodies (EBs) remains a major challenge, and the current lack of standardization and comparability is still limiting a broader application and translation of stem cell technology. Among others, a vital aspect for the reliable formation of EBs is optimizing differentiation protocols since organized differentiation is influenced by soluble inducers and EB size. A microfluidic biochip array was employed to automate cell loading and optimize directed neuronal and astrocytic differentiation protocols using murine P19 embryoid bodies to facilitate reliable embryonic stem cell differentiation. Our gravity-driven microfluidic size-controlled embryoid body-on-a-chip system allows (a) the robust operation and cultivation of up to 90 EBs in parallel and (b) the reproducible generation of five increasing sizes ranging from 300 µm to 1000 µm diameters. A comparative study adds two differentiation-inducers such as retinoic acid and EC23 to size-controlled embryoid bodies to identify the optimal differentiation protocol. Our study revealed a 1.4 to 1.9-fold higher neuron and astrocyte expression in larger embryoid bodies (above 750 µm) over smaller-sized EBs (below 450 µm), thus highlighting the importance of EB size in the establishment of robust neurodevelopmental in vitro models.

## 1. Introduction

The global trend towards improved in vitro three-dimensional tissue models in biomedical research has led to an increased application of advanced stem cell technologies using iPSC- and embryonic stem cells. Due to their inherent pluripotency and self-renewal capacity, embryonic stem cells (ESCs)–the inner cell mass of a blastocyst–provide an indispensable and powerful tool in basic and applied research. By cultivating embryonic stem cells in three-dimensional (3D) cellular aggregates, early stages of embryonic development can be recapitulated in vitro in the form of so-called embryoid bodies (EBs). These in vitro derived structures give rise to all three primary germ layers, including the ectoderm, the mesoderm, and the endoderm [1,2,3]. Their ability to emulate complex embryonal micro-tissues in vitro, including structures of the central nervous system, make embryoid bodies ideally suited in the field of neuroscience since several neurological disorders such as autism, schizophrenia, and microencephaly are of neurodevelopmental origin [4]. Moreover, the study of early embryonic events is of particular importance to gain deeper insights into processes that influence cell fate decisions ranging from cell-to-cell interactions, cell communication such as neuralizing signals, biochemical cues, and systemic circulatory factors such as growth factors, etc. [5,6,7,8].

Despite this tremendous translational potential of embryonic stem cell technology, many technical challenges still persist, including the reproducible generation of identical embryoid bodies, a fact that still hampers their mainstream application and limits their therapeutic power. In other words, a significant challenge in achieving homogeneous lineage-specific differentiation from heterogeneous embryoid bodies to date is associated with the inability to properly control cellular microenvironments [9,10,11]. In addition to numerous soluble factors that govern differentiation, mounting scientific evidence has pointed at an embryoid body size-dependent differentiation capacity of stem cells in recent years. This means that any inconsistencies in embryoid body size need to be addressed since embryonic stem cell position inside the embryoid body also governs cellular fate decisions. Therefore, it has been suggested that the spatially distinct microenvironment within the embryoid body directly alters cell-to-cell interactions during the differentiation process [12,13,14,15]. Indeed, a handful of publications have already indicated an embryoid body size-dependent effect on the microtissues fate; however, due to the limited number of studies, the results remain at best inconsistent and inconclusive [16,17,18]. Consequently, variabilities of experimental protocols will lead to different embryoid body sizes, which in turn demand time-consuming protocol validations and costly trial-and-error optimization studies to yield the cell type of interest [19,20]. In recent years, various miniaturized methods have been investigated to control the homogeneity of embryoid body size and shape, including microwell-based designs [21,22,23,24] and micropatterning techniques such as micro stencils and conventional methods soft lithography and microcontact printing [25,26,27]. However, to effectively assess all critical factors concerning their ability to promote embryonic stem cells to differentiate into the desired cell types, robust, reliable, and reproducible embryoid body technologies are needed. 

One cell culture technology capable of precisely regulating microenvironments of stem cells is called organ-on-a-chip technology, where 3D-cell assemblies, spheroids, and organoids can be cultured inside microfluidic devices under near-physiological conditions [28,29,30,31,32,33]. It is important to highlight that to test a large number of embryoid bodies in a reproducible and high-content manner; the following criteria need to be addressed:

(1) optimal cultivation conditions over several days,
(2) precise control of nutrient and gas exchange,
(3) user-friendly cell loading capacities,
(4) parallel embryoid body production and harvesting option,
(5) simple operation to ensure tissue maintenance.

**Figure 1:**
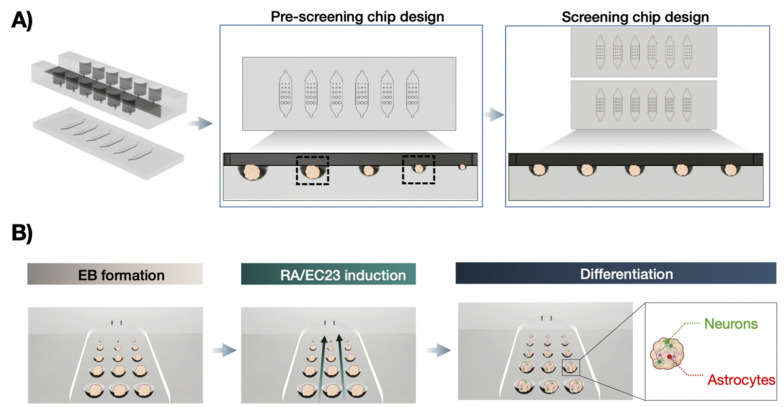
(**A**) Microfluidic biochip array concepts using a pre-screening chip design of multiple cavity sizes for embryoid body (EB) size optimization and two screening chip designs consisting of a single microcavity size on each individual chip. (**B)** Workflow of on-chip differentiation protocol including EB formation, exposure with neuralizing inducers retinoic acid (RA) or EC23, followed by differentiation in neurons and astrocytes. Created with BioRender.com.

In this study, we have employed a microfluidic embryoid body-on-a-chip array to study, in detail, the effects of embryoid body size on both neuronal and glial differentiation. Additionally, the cooperative impact of the two chemical inducers retinoic acid (RA) and its photostable synthetic analog EC23, are tested in a dose-dependent manner over a differentiation period of 14 days. Retinoic acid is chosen in this study because it is essential in the differentiation process of the adult hippocampus and has long been recognized as a robust neuronal differentiation-inducing molecule in vitro [34,35,36,37]. Embryoid bodies are generated using the embryonal carcinoma cell line P19, which is well-known as an excellent model to study embryonic stem cell maintenance and differentiation. In an initial pre-screening study, optimal P19 embryoid body dimensions are investigated using a multi-sized biochip array capable of simultaneously generating up to 90 spheroids of five sizes on a single platform (see Figure 1A). In a subsequent series of experiments, the influence of increasing concentrations of RA and EC23 on embryoid body differentiation is assessed using two pre-selected embryoid body sizes. Here, the two single-sized microfluidic spheroid array platforms comprising six microfluidic channels, each containing 15 identical hemispherical microwells, were used for embryoid body generation [38]. Medium perfusion is accomplished by moving fluids between two media reservoirs using a rocking shaker (see Figure S1). Using this microfluidic configuration shown in Figure 1B enables the reliable generation and dynamic cultivation of size-controlled embryoid bodies. It allows for parallel screening of several inducer concentrations and time-resolved monitoring of several embryoid body sizes.

## 2. Materials and Methods

### 2.1. Cell Culture

The murine embryonal carcinoma cells line P19 (ATCC, Manasass, VA, USA, CRL-1825) was maintained in Minimum Essential Medium Alpha Modification (α-MEM; Sigma-Aldrich, Vienna, Austria) supplemented with 7.5% newborn calf serum (Sigma-Aldrich, Vienna, Austria), 2.5% fetal bovine serum (Sigma-Aldrich, Vienna, Austria), and 1% antibiotic/antimycotic solution (Sigma-Aldrich, Austria) under standard cell culture conditions at 37 °C in a 5% CO_2_ humidified atmosphere. The medium was changed every three days, and cells were passaged at 90% confluency. After a washing step with Dulbecco’s Phosphate-Saline Buffer (DPBS; Sigma-Aldrich, Vienna, Austria), cells were enzymatically detached with 3 mL 0.25% Trypsin-EDTA (Sigma-Aldrich, Vienna, Austria) and subsequently pelleted at 140 × g for five minutes. The supernatant was aspirated, and the cell suspension was adjusted to required cell densities with a complete α-MEM medium. P19 cells were passaged twice or three times per week at a ratio of 1:4–1:6.

### 2.2. Microfluidic Chip Fabrication

As described previously, the master mold and microfluidic biochip array fabrication were conducted [38]. In brief, a 1:10 polydimethylsiloxane mixture (PDMS; Sylgard 184 Silicon, Farnell, Austria) was degassed to remove air bubbles and poured into the two different molds a lower cell culture chamber mold and the upper reservoir mold. The microwell array layers were cured at 80 °C and the upper reservoir layers at 70 °C for two hours. For assembly, PDMS surfaces plasma-activated for 30 s at 0.6 mbar and 200 W (Diener, Ebhausen, Germany), aligned, and bonded at 80 °C overnight.

### 2.3. Microfluidic Chip Priming and Cell Seeding

Chips were initially coated with Lipidure® CM52006 (AMSbio, Abingdon, UK) using 100 µL ethanolic Lipidure® solution to induce EB formation and inhibit cell outgrowth. After evaporation at 80 °C, chips were filled with 70% ethanol and subsequently exchanged washed three times with PBS supplemented with 1% antibiotic/antimycotic solution (Sigma-Aldrich, Vienna, Austria) under sterile conditions. Finally, the channels were flushed twice with cell culture media and filled with P19 cell suspensions in a complete α-MEM medium. Chips were maintained and incubated at 37 °C in a 5% CO_2_ humidified atmosphere and placed on a rocker platform set to a flow rate of 4 μL/min at 1° tilting angle and 1 rpm with medium exchange every two days. 

### 2.4. Viability Analysis

A dye exclusion assay based on a solution containing 1.0 mg/mL Hoechst 33,342 (Invitrogen, Thermo Scientific, Vienna, Austria) and 2.0 mM ethidium bromide homodimer-1 (Sigma-Aldrich, Vienna, Austria) in PBS was performed to determine cell viability. EBs were rinsed with 200 µL of the staining solution two times and incubated for 30 min at 37 °C. To avoid photobleaching, all steps were done in the dark. Fluorescence signals were analyzed by fluorescence imaging on an IX83 live-cell microscope setup (Olympus, Hamburg, Germany) using DAPI (ex. 390 nm, em. 460 nm) and TRITC filter sets (ex. 530 nm, em. 645 nm). 

### 2.5. P19 Induction and Embryoid Body Differentiation

After three days post-seeding, EBs were treated with 0.5 µM, 1.0 µM, and 10.0 µM of RA (Sigma-Aldrich, Austria) or EC23 (Sigma-Aldrich, Vienna, Austria) for two or four days. After exposure, media was changed to neuronal differentiation medium containing 1× Neurobasal Medium (Gibco, Thermo Fisher, Vienna, Austria), 2% B27 supplement (Gibco, Thermo Fisher, Vienna, Austria), 0.5 mM L-Glutamine (Gibco, Austria) and 1% antibiotic/antimycotic solution (Sigma-Aldrich, Vienna, Austria) and exchanged every second day. For static experiments, 200 µL of 5000 cells/mL were seeded in 96-ultralow attachment plates (Corning, Vienna, Austria) and treated as described previously. After two- and four-day induction, the EBs were transferred to 0.1% gelatin-coated microscope slides (Sigma-Aldrich, Vienna, Austria) with 18 mm well sizes (0.5 mm chamber height). 

### 2.6. Immunofluorescence Imaging

For immunofluorescence staining, the EBs at day one, seven, and 14were fixed with 4% paraformaldehyde in Dulbecco’s phosphate-buffered saline (DPBS^+^; with MgCl_2_ and CaCl_2_, Sigma-Aldrich, Vienna, Austria) overnight at 4 °C. The next day, EBs were washed with DPBS^+^ and permeabilized with 0.2% Triton X-100 (Sigma-Aldrich, Vienna, Austria) for 15 min at RT. After blocking for two hours with 1% bovine serum albumin (BSA; Sigma-Aldrich, Vienna, Austria), primary antibodies for neuronal microtubule-associated protein 2 (MAP2) and astrocytic glial fibrillary acidic protein (GFAP) were diluted in blocking solution 1:1000 and 1:250 and incubated at 4 °C overnight. Specimens were washed twice for 15 min with DPBS^+^ on a shaker platform at 600 rpm (VWR, Vienna, Austria) and incubated with goat anti-rabbit 555 (1:1000; Abcam, UK) and goat anti-chicken 488 secondary antibody (1:1000; Abcam, Cambridge, UK) solution for one hour at RT. Samples were washed twice for 15 min with DPBS^+,^ and nuclei were counterstained for one hour with 2 mg/mL DAPI (Sigma-Aldrich, Vienna, Austria) diluted in DPBS^+^. After a final DPBS^+^ washing step, samples were transferred onto a microscope slide and mounted with a Vectashield^®^ soft embedding mounting medium (Vector Laboratories, SZABO-SCANDIC HandelsGmbH, Vienna, Austria). 

### 2.7. General Microscopy and Morphometric Analysis

To investigate spheroid size and morphology, bright-field images were taken by using an IX83 microscope (Olympus, Vienna, Austria) equipped with temperature, CO_2_, and O_2_ control (Peacon, Erbach, Germany) and a high-resolution ORCA-Flash 4.0 camera (Hamamatsu, Hamamatsu, Japan). For imaging the whole cultivation channel, MIA scans were conducted using 4× and 10× magnification. All images were processed by ImageJ (Version 1.52, NIH, Bethesda, MD, USA) to quantify the spheroidal diameter of the embryoid bodies. 

For viability determination of embryoid bodies, image backgrounds were subtracted (rolling ball subtraction), and mean fluorescence intensities of embryoid bodies were calculated as described in Equation (1):

(1) $$Viability\;\left(\%\right)=100-\left(\frac{FI\;Ethidium}{FI\;Hoechst}*100\right)$$

To evaluate the differentiation pattern, the fluorescence intensities of the specific antibodies were set in relation to DAPI. For that, the image channels of each picture were merged, and the signals were measured by using the built-in RGB measure plugin of ImageJ. For morphometric analysis of neuronal and astrocytic networks of EBs, AngioTool (Version 0.6a) was used with optimized analysis parameters (thickness: 12; low-intensity threshold: 24–26; remove small particles option set to 603).

### 2.8. Statistical Analysis

Statistical significance was tested using Student’s t-test, ordinary one-way ANOVA, two-way ANOVA, or mixed-effect analysis performed by GraphPad Prism 8 (Version 8.2.1, GraphPad, San Diego, CA, USA). *p* values < 0.05were considered as statistically significant (* *p* < 0.05, ** *p* < 0.01, *** *p* < 0.001). The data are presented as the mean of experiments ± standard deviation (SD).

## 3. Results and Discussion

### 3.1. Influence of RA and EC23 Concentration on Embryoid Body Formation

**Figure 2:**
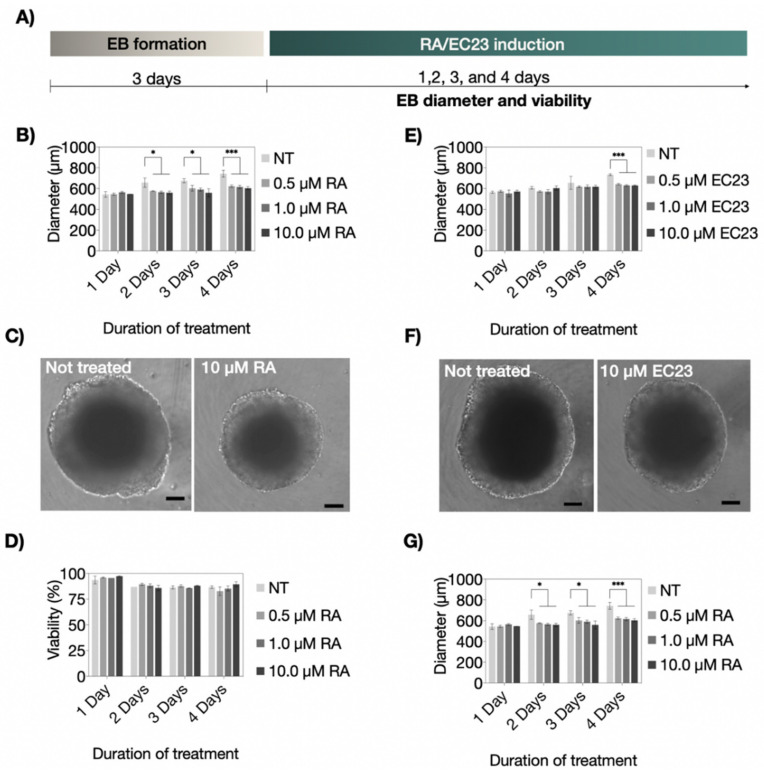
(**A**) Schematic workflow of neuronal and astrocytic differentiation protocols using retinoic acid (RA) or EC23 as inducers. Diameters of embryoid bodies after one-, two-, three- and four-day treatment with concentrations of non-treated (NT), 0.5 µM, 1.0 µM, and 10 µM of (**B**) RA and (**E**) EC23. Bright-field micrographs of non-treated, (**C**) 10 µM RA-treated, and (**F**) 10 µM EC23-treated P19 embryoid bodies in a microtiter plate after four-day treatment. Scale bar, 100 µm. Viability of embryoid bodies after one-, two-, three- and four days post-treatment with increasing concentrations of (**D**) RA and (**G**) EC23. Statistical significance was tested by using one-way ANOVA (* *p* < 0.05 and *** *p* < 0.001), *n* = 3 ± SD. Bars without * do not represent statistical significance.

Although the Vitamin A derivatives RA and EC23 are commonly used for triggering stem cell differentiation to a neuronal phenotype [39], elevated levels of both molecules are known to induce neurotoxic effects [40,41]. Consequently, as indicated in Figure 2A, an initial cultivation optimization study was performed to evaluate the dose-dependent effects of RA and EC23 on embryoid body viabilities. Potential cytotoxicity for increasing concentrations of RA and EC23 (e.g., 0.5, 1.0, and 10.0 µM) was measured by comparing P19 embryoid body morphology and viability over a cultivation period of up to four days of induction. Results shown in Figure 2B–D revealed stable embryoid body diameters independent of the RA concentration throughout the entire four days of induction. Time-dependent embryoid body diameters are shown in Figure 2B,C yielded initial values of 540.7 ± 11.7 µm, 558.1 ± 8.6 µm, and 546.4 ± 0.5 µm at day 1, and 631.4 ± 10.7 µm, 619.8 ± 15.8 µm, and 595.6 ± 15.0 µm at day 4, respectively. In contrast, untreated embryoid body sizes increased by 30% over four days resulting in maximum diameter of 734.1 ± 33.6 µm. Next, embryoid viabilities are closely monitored during a four-day induction period to exclude cytotoxic events caused by RA treatments that may reduce embryoid body diameter. As shown in Figure 2D, no cytotoxic effects of RA (up to 10.0 µM RA) were detectable. All treated and untreated embryoid bodies exhibited comparable viability values in the range of 82–98%. This result is in line with previous observations and points at a significant reduction in the proliferation capacity of embryonic stem cells when transitioning from a proliferative to a cell-differentiation stage [42]. Interestingly, a reduced dose-dependent size effect was found in EC23 treated embryoid bodies, thus pointing at a limited induction capacity. Figure 2E,F reveals no significant differences between treated and untreated embryoid bodies until day 4 of induction. Similarly, EC23 treatment (up to 10.0 µM) did not induce any significant cytotoxic effects on embryoid body viability over a treatment period of 4 days. Results in Figure 2G show average cell viability in the range of 79–96% between treated and untreated samples.

### 3.2. Neuronal and Astrocyte Differentiation Capacity of RA and EC23 Treated EBs

**Figure 3:**
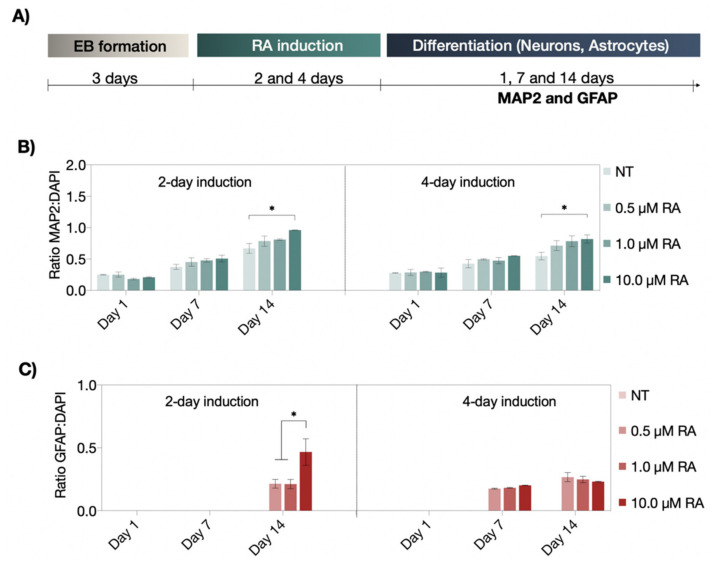
(**A**) Schematic workflow of the neural differentiation protocol of RA-induced neuronal and astrocytic differentiation. (**B**,**C**) Time-resolved expression of neuronal (MAP2; green fluorescence) and astrocytic (GFAP; red fluorescence) differentiation markers after two- and four-day treatment with concentrations of non-treated (NT), 0.5 µM, 1.0 µM, and 10 µM of RA under static cultivation condition. Statistical significance was tested by using one-way ANOVA (* *p* < 0.05), *n* = 2 ± SD. Bars without * do not represent statistical significance.

Following the verification of suitable embryoid body viabilities, the ability of RA and EC23 to induce P19-stem cell differentiation into neurons and astrocytes was investigated in subsequent sets of experiments. Figure 3A shows MAP2 and GFAP-expression results indicating the presence of either neurons or astrocytes after two- and four-day induction protocols. Here, the normalized MAP2 signal—an indicator of neuronal differentiation—was monitored over a maturation period of up to 14 days after induction. While both two- and four-day RA-induction protocols exhibited a significant increase in MAP2:DAPI signal over time, no significant differences were observed within the first seven days post-induction, independent of exposure duration and RA-concentration. However, the two-day exposure scenario resulted in a 1.2-fold higher number of MAP2-positive neurons than the four-day protocol after 14 days post-induction. Moreover, the strongest neuronal differentiation was identified for embryoid bodies treated with 10.0 µM RA using the shorter two-day induction period. In turn, for astrocytic differentiation, clear differences between two- and four-day induction protocols were observed. For instance, while a two-day RA-induction led to a GFAP expression increase only after 14 days of differentiation, the longer four-day induction protocol resulted in the presence of astrocytes already after one-week post-induction (see Figure 3C). Similarly, the strongest astrocyte differentiation was observed at 10 µM RA, exhibiting a 2.2-fold higher GFAP intensity than any other induction strategy. These results highlight the difficulties when using biochemical inducers to generate optimum differentiation protocols. 

**Figure 4:**
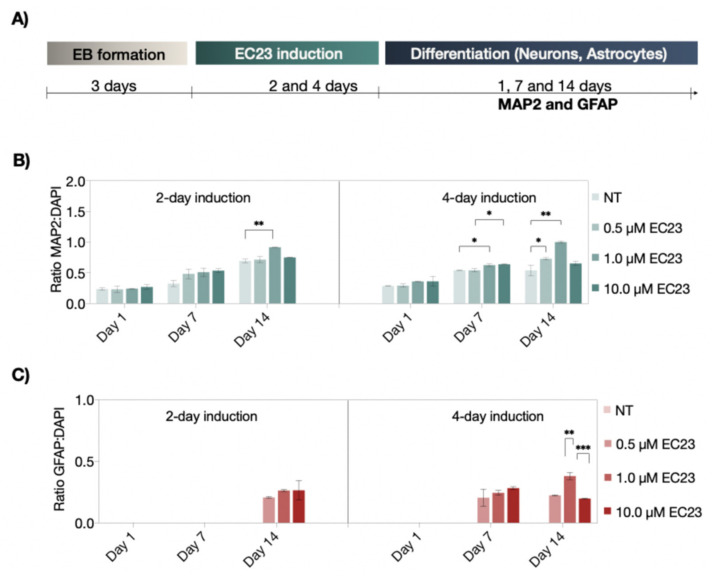
(**A**) Schematic representation of the neural differentiation protocol of EC23-induced neuronal and astrocytic differentiation. (**B**,**C**) Time-resolved expression of neuronal (MAP2; green) and astrocytic (GFAP; red) differentiation markers after two- and four-day treatment with concentrations of non-treated (NT), 0.5 µM, 1.0 µM, and 10 µM of EC23 under static cultivation condition. Statistical significance was tested by using one-way ANOVA (* *p* < 0.05, ** *p* < 0.01, and *** *p* < 0.001), *n* = 2 ± SD. Bars without * do not represent statistical significance.

Similar to RA, the photostable analog EC23 resulted in a time-resolved increase in MAP2 positive cells, independent of the induction strategy. However, strong concentration-dependent effects were observed in embryoid bodies treated with 10 µM EC23, revealing no significant differences between untreated and treated EBs at day 14 of differentiation for both induction scenarios, indicating a RA receptor saturation effect (see Figure 4A–C). Interestingly, astrocyte differentiation was already detected after 7 days only when using the four-day induction protocol. 

**Figure 5:**
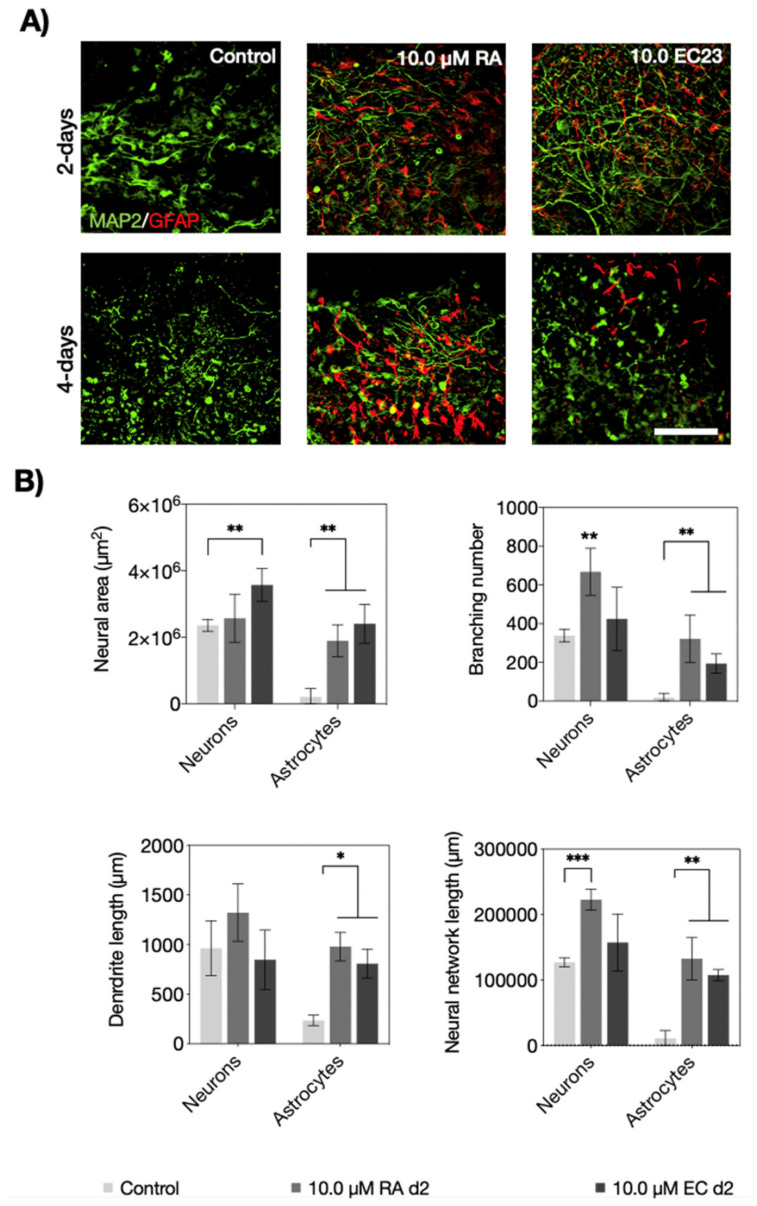
(**A**) Fluorescence micrographs of neurons (MAP2; green) and astrocytes (GFAP; red) after two-and four-day treatment with 10.0 µM retinoic acid (RA) and EC23 and 14-days of differentiation. Scale bar, 100 µm. (**B**) Quantification of neural area, branching number, process lengths, and network length of neuronal and astrocytes after two-day treatment with 10.0 µM retinoic acid (RA) and EC23 and 14-days of differentiation. Statistical significance was tested by using one-way ANOVA (* *p* < 0.05, ** *p* < 0.01, and *** *p* < 0.001), *n* = 3 ± SD. Bars without * do not represent statistical significance.

Next, neural morphometric parameters including network area, degree of branching, process length, and overall network length were investigated for both inducers. Figure 5 shows the results for RA and EC23 at the highest concentrations of 10 µM. For instance, fluorescence images shown in Figure 5A confirmed that both the induction period and the inducer type influence neuronal and astrocytic network morphologies of P19-derived embryoid bodies. Initial comparison between the two-day induction protocols revealed that the neuronal area was significantly improved for EC23 (*p* < 0.01) but not for RA, while astrocytes responded similarly for both inducers (*p* < 0.01). Additionally, neuronal branching was improved significantly in the presence of RA (*p* < 0.0021), and both treatment strategies improved astrocytic branching compared to the untreated control embryoid bodies (*p* < 0.01). Moreover, process length was significantly increased only for astrocytes and not neurons (*p* < 0.05). Furthermore, neural network length was significantly pronounced for neurons when embryoid bodies were treated with RA *p* < 0.001). A similar comparative analysis between neural morphology parameters of embryoid bodies is shown in Figure S2 and revealed that both inducers improved astrocytic parameters alike. For instance, a 126- and 128-fold increase of astrocytic network area, 331- and 300-fold increase of astrocyte branching, 2.1- and 2.8-fold increase of astrocytic process length was found, as well as an overall improvement of 130-fold of total astrocytic network length (longest interconnected path) for both 10 µM RA and EC23, respectively. Overall, these results indicate that higher concentrations of 10 µM RA and EC23 tend to improve neuronal differentiation, whereas elongation of the induction period stimulates especially astrocytic differentiation in P19 embryoid bodies. 

### 3.3. Optimization of P19 Cell Seeding Protocols for Size-Controllable On-Chip Embryoid Body Formation

**Figure 6:**
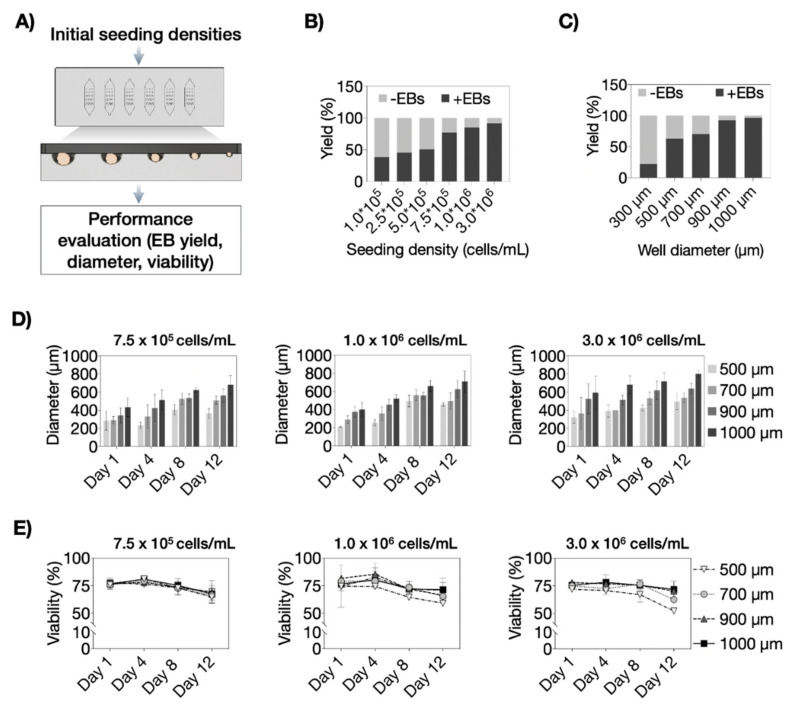
(**A**) Embryoid body (EB) size pre-screening concept using multi-size biochip array by evaluating EB yield, EB diameter, and EB viability. Average EB production yield at respective (**B**) initial seeding densities and (**C**) biochip well diameters during a cultivation period of 12 days in the microfluidic array. (**D**) EB size separation by different microwell diameters (500–1000 µm) at initial seeding densities of 7.5 × 10^5^ cells/mL, 1.0 × 10^6^ cells/mL, and 3.0 × 10^6^ cells/mL for 12 days post-seeding in the microfluidic device. Statistical significance was tested by using one-way ANOVA, *n* = 3 ± SD. Bars without * do not represent statistical significance. (**E**) Time-resolved monitoring of the impact of initial seeding densities and microwell sizes on EB viability, *n* = 3 ± SD.

An initial cell seeding optimization study was conducted to identify the ideal P19 cell density needed to reproducibly load each microwell with identical cell numbers inside the microfluidic biochip array. Figure 6A shows the experimental setup where increasing numbers of P19 embryonic stem cells were loaded, allowed to form cell aggregates of different sizes, and subsequently analyzed to estimate embryoid body yield, growth, and viability. The first critical biochip parameter results are shown in Figure 6B, indicating that embryoid body yield remained below 50% during the cultivation period of 12 days using an initial seeding density of 1.0 × 10^5^, 2.5 × 10^5^, and 5.0 × 10^5^ cells/mL. However, increasing cell densities of 7.5 × 10^5^ cells/mL, 1.0 × 10^6^ cells/mL and 3.0 × 10^6^ cells/mL produced excellent yields of 91 ± 9%, 98 ± 5% and 96 ± 6%, respectively. Interestingly, the overall lower average yield of ca. 90% using a seeding density of 7.5 × 10^5^ cells/mL was caused by limited ability to generate embryoid bodies (e.g., 22% yield) inside the smallest microwells of 300 µm diameters, as shown in Figure 6C, and was therefore excluded for next experiments. Next, the influence of higher cell seeding densities on cell proliferation was investigated to verify the generation of highly viable embryoid bodies. Figure 6D shows growth characteristics of four increasing embryoid body sizes over a 12 days of on-chip cultivation period (*p* > 0.01) using three seeding densities of 7.5 × 10^5^ cells/mL, 1.0 × 10^6^ cells/mL, and 3.0 × 10^6^ cells/mL. While no discernable influence of seeding density on embryoid body growth was detected, cell viability was affected in the presence of high cell seeding densities over time. As an example, using the highest seeding density of 3.0 × 10^6^ cells/mL yielded the lowest and gradually stronger declining viability values, particularly in smaller spheroid sizes and significant size-dependent differences (*p* < 0.05), while the lower 7.5 × 10^5^ cells/mL did not show any toxicities (*p* = 0.49) independent of cultivation time and increasing embryoid body sizes. Notably, the lowest initial seeding density also showed better reproducibility and size control throughout the entire cultivation period. Consequently, the final set of experiments covering on-chip neuronal and astrocytic differentiation analysis using various embryoid body sizes was conducted using an initial cell seeding density of 7.5 × 10^5^ cells/mL. 

### 3.4. On-Chip Screening of Embryoid Body Size Effects on Neuronal and Astrocytic Differentiation Capacity

**Figure 7:**
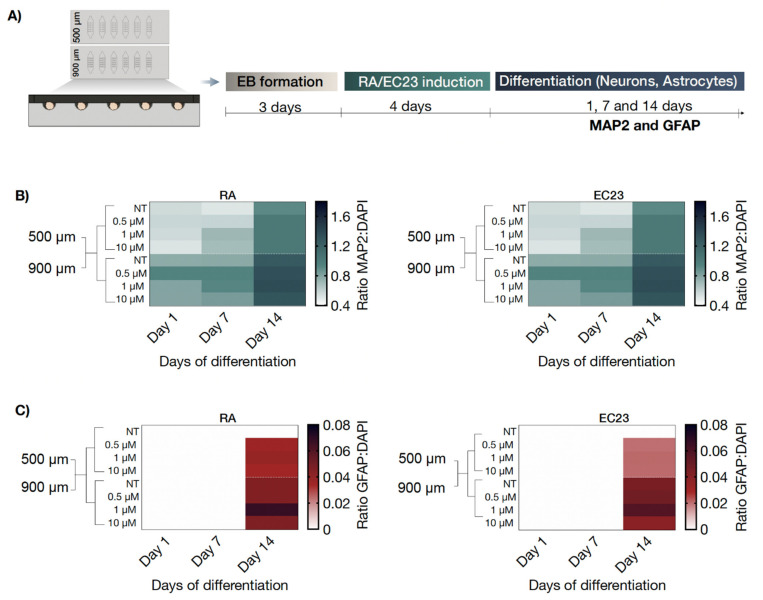
(**A**) Workflow of the microfluidic differentiation protocol by using single-sized microfluidic biochip arrays (500 and 900 µm well diameters) including on-chip EB generation, exposure to retinoic acid (RA) and EC23, as well as differentiation into neurons and astrocytes. Influence of two EB sizes on (**B)** neuronal (MAP2, green) and (**C**) astrocytic (GFAP; red) differentiation marker expression relative to cell nuclei (DAPI) during 14 days after a 4-day exposure of RA or EC23 and 14 days of differentiation, *n* = 1 ± SD.

In a final set of experiments, embryoid body size-related effects on neural and astrocytic differentiation were monitored over a total cultivation time of 14 days post-induction with either RA or EC23 inducers. As shown in our differentiation optimization studies (see Figure 3 and Figure 4), a four-day induction period induces earlier astrocytic differentiation upon stimulation was chosen as the standard protocol for all remaining on-chip experiments. To investigate the impact of embryoid body size on neural differentiation, embryoid bodies were generated inside biochips containing representative sizes of 500 µm and 900 µm-diameter microcavities (see also Figure 7A). Results of our comparative analysis are shown in Figure 7B and revealed a gradual increase in MAP2 positive neurons over two weeks, as indicated by a 1.7 ± 0.1-fold change of MAP2 signal for RA (*p* < 0.001) and 1.6 ± 0.2 (*p* < 0.001) fold change for EC23 at day 14 for 500 µm wells. Similar concentration-independent effects were observed for embryoid bodies that have been generated in 900 µm sized microcavities (*p* < 0.05 for RA and EC23). It is important to highlight that embryoid bodies generated and differentiated within 900 µm microcavity displayed significantly higher MAP2 levels than the smaller 500 µm embryoid body size, thus confirming the direct influence of size on cellular differentiation status. In turn, untreated embryoid bodies cultivated in 500 µm microcavities showed no GFAP signal and completely lacked any astrocyte cell population. In contrast, RA and EC23-treated embryoid bodies showed a significant concentration-independent increase of astrocytes (*p* < 0.01), as shown in Figure S3. These results point at additional spontaneous astrocytic differentiation only in larger-sized embryoid bodies. 

## 4. Conclusions

Embryoid bodies serve as an advanced cell-based tool to treat congenital neural disorders and regenerate neural tissues lost as a result of neurodegenerative diseases and injuries [43]. However, to realize their full potential for biomedical research, clinical routine and industrial applications, pluripotent stem cells are needed for scalable and reproducible differentiation protocols. To date, however, appropriate differentiation protocols have not been developed to reliably produce large amounts of defined embryoid bodies. The main limitation of standardization and harmonization of embryoid body differentiation protocols is associated with the unpredictable formation of various differentiated cell types, thus leading to great variability in differentiation status. Among the types of multipotent stem cells, it has been shown that embryonic stem cells can differentiate into neurons, astrocytes, and oligodendrocyte lineages in vitro [44]. 

One biophysical parameter that affects the differentiation efficiency and rate of embryonic stem cells is linked to the embryoid body size and its three-dimensional architecture [45], thereby making size-control an important factor in developing optimized embryoid body differentiation protocols. It is, however, important to note that size uniformity and productivity of stem cell aggregates are known to be a trade-off relationship [46]. For instance, concentration gradients of molecules such as oxygen, nutrients, and metabolites vary as a function of embryoid body size and consequently affect the proliferation and maturation of stem cells. Additionally, the initial cell numbers of embryoid bodies are considered a critical factor for directed differentiation [47]. To find the ideal balance between embryoid body size and differentiation capacity of embryonic stem cells, a size-controlled embryoid body-on-a-chip system was used to establish more reproducible and controllable differentiation protocols for producing a large amount of uniformly sized embryoid bodies. Our findings confirmed that precise control over embryoid body size leads to a reproducible and well-defined cell culture system. In contrast, the ability to generate various embryoid body sizes inside our microfluidic biochip array allows for identifying optimal neural differentiation rates of embryonic stem cells. Results of our study have shown that embryoid body size affects long-term viability and morphology and directly influences the differentiation of neurons and astrocytes in vitro. For instance, larger EBs exhibited higher spontaneous astrocyte differentiation capacity, while smaller clusters differentiated in a controlled manner with increasing inducer concentrations. On the other hand, the highest neuronal and astrocytic signals were achieved in larger embryoid bodies, showing the challenges and need for optimization tools to find the optimal balance between EB size, viability, incubation time, and directed differentiation capacity. In our study, the microfluidically optimized EB protocol includes the generation of EBs of 450 µm in diameter (generated in 500 µm microwells) as the best-suited size for directed differentiation after 4-day exposure with RA or EC23 followed by a differentiation period of 14 days since larger EBs lack controlled differentiation capacity. This was demonstrated by the spontaneous formation of astrocytic structures in large EBs even in the absence of differentiation inducers in contrast to smaller non-treated EBs that showed no GFAP signals.

In conclusion, this platform demonstrates a facile approach to manipulate embryoid body size and their microenvironment enabling new opportunities to efficiently adapt stem cell culture conditions and direct cell fate for robust neurodevelopmental in vitro studies.

## Data Availability

Data are available under reasonable email request.

## Supplementary Materials

The following supporting information can be downloaded at: www.mdpi.com/article/10.3390/pharmaceutics14020339/s1, Figure S1: Schematic drawing of the three microfluidic spheroid array designs with (A) top and side view of the microcavitiy arrays, and (B) top and front view on the microarray slide. Figure S2: Comparative morphometric analysis of neuronal and astrocytic parameters including (A) area, (B) branching, (C) process length, and (D) network length for EBs generated with 2- and 4-day RA and EC23 induction protocols, *n* = 3 ± SD. Figure S3: Immunofluorescence micrographs of neurons (MAP2, green) and astrocytes (GFAP; red) after 4-day exposure to retinoic acid (RA) or EC23 and 14 days of differentiation of small (500 µm) and large (900 µm) embryoid bodies on-chip. Scale bar, 100 µm.
