# Supplementary material for: Screening for Best Neuronal-Glial Differentiation Protocols of Neuralizing Agents Using a Multi-Sized Microfluidic Embryoid Body Array
Source: Pharmaceutics. 2022 Jan 31;14(2):339. doi: 10.3390/pharmaceutics14020339 (PMC8878393; doi:10.3390/pharmaceutics14020339)
Supplement: Supplementary file 1 [file pharmaceutics-14-00339-s001.zip › pharmaceutics-1542122-supplementary.pdf]

pharmaceutics

Supplementary Materials: Screening for Best Neuronal-Glial Differentiation Protocols of Neuralizing Agents Using a Multi-Sized Microfluidic Embryoid Body Array

Christoph Eilenberger, Mario Rothbauer, Konstanze Brandauer, Sarah Spitz, Eva-Kathrin Ehmoser, Seta Küpcü and Peter Ertl

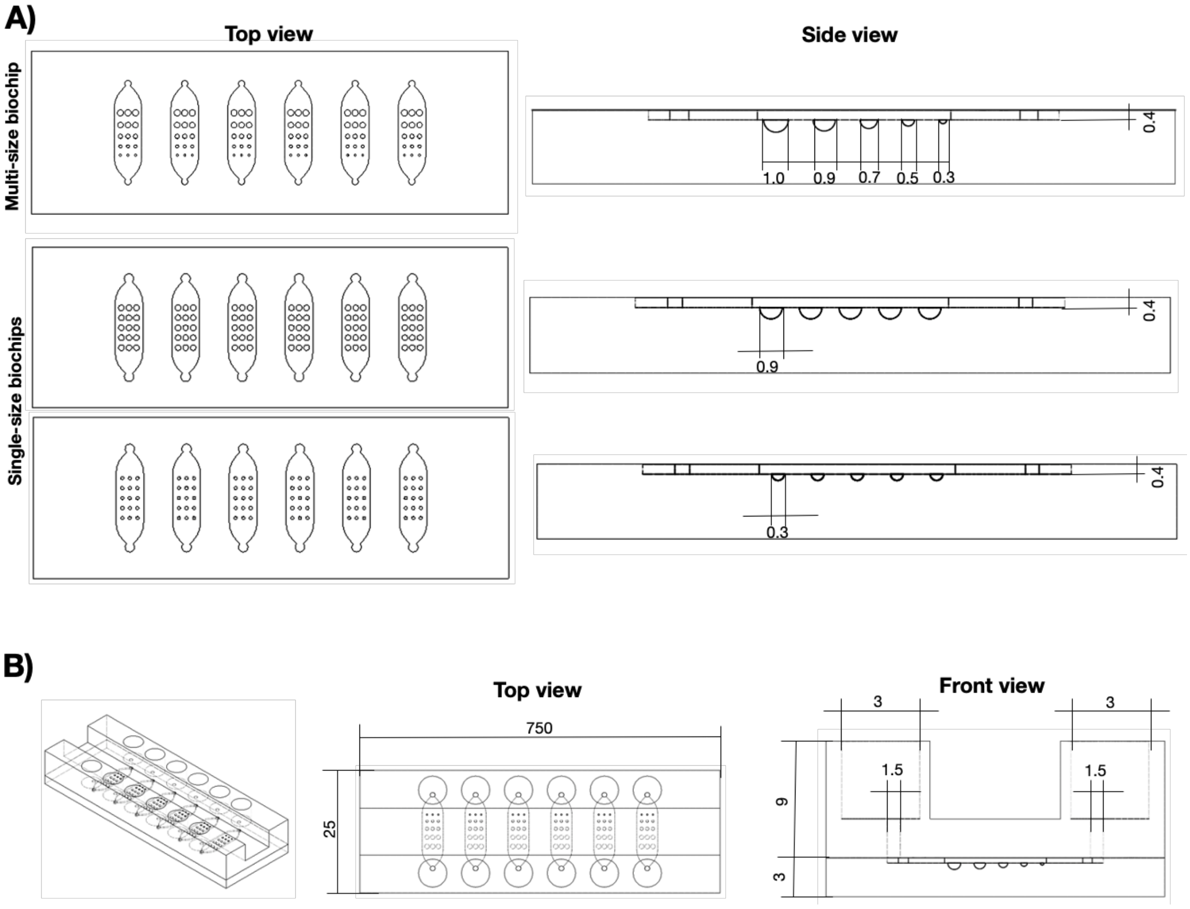

Figure S1. Schematic drawing of the three microfluidic spheroid array designs with (**A**) top and side view of the microcavitiy arrays, and (**B**) top and front view on the microarray slide.

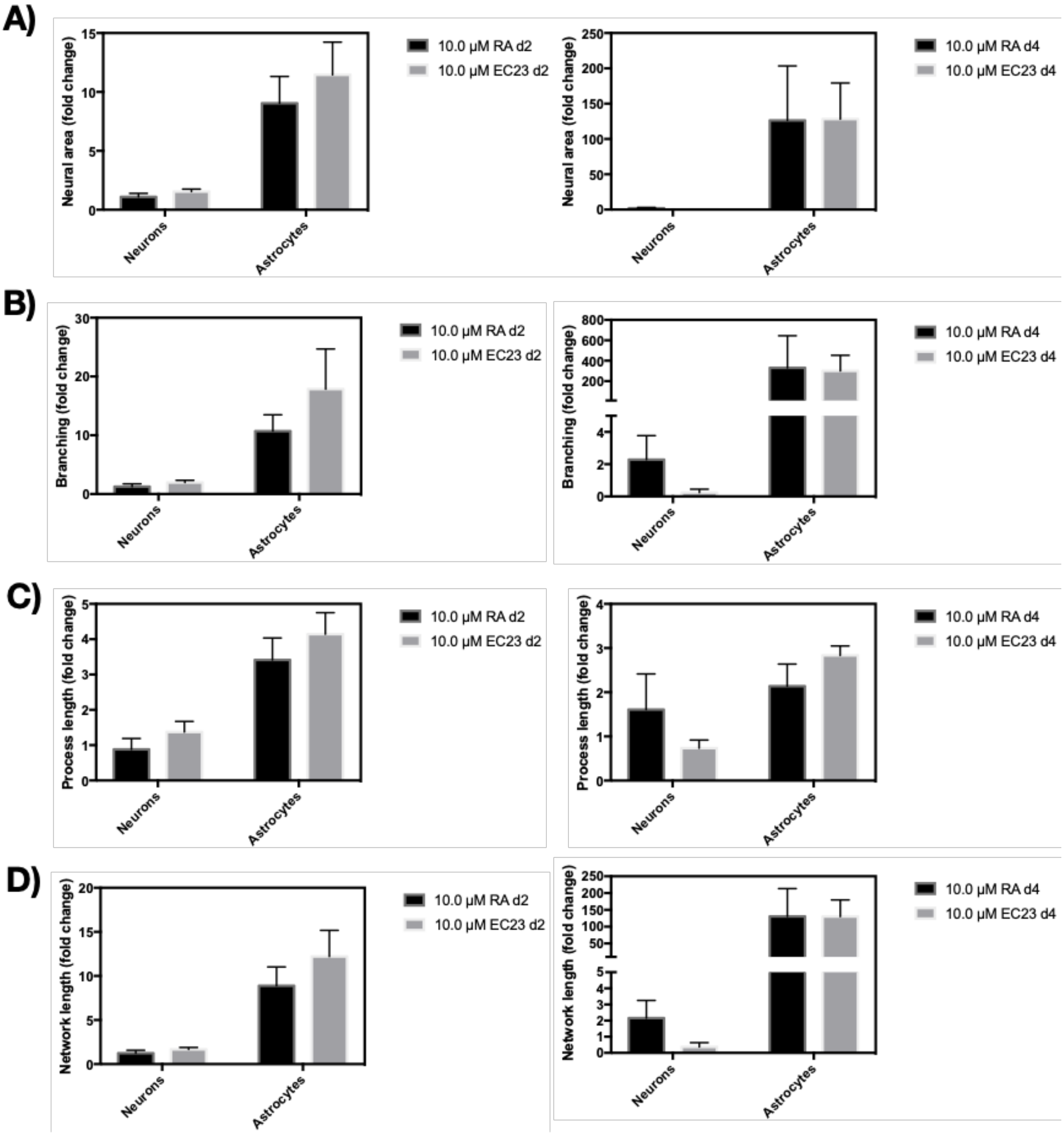

Figure S2. Comparative morphometric analysis of neuronal and astrocytic parameters including (**A**) area, (**B**) branching, (**C**) process length, and (**D**) network length for EBs generated with 2- and 4-day RA and EC23 induction protocols, $n = 3 \pm SD$.

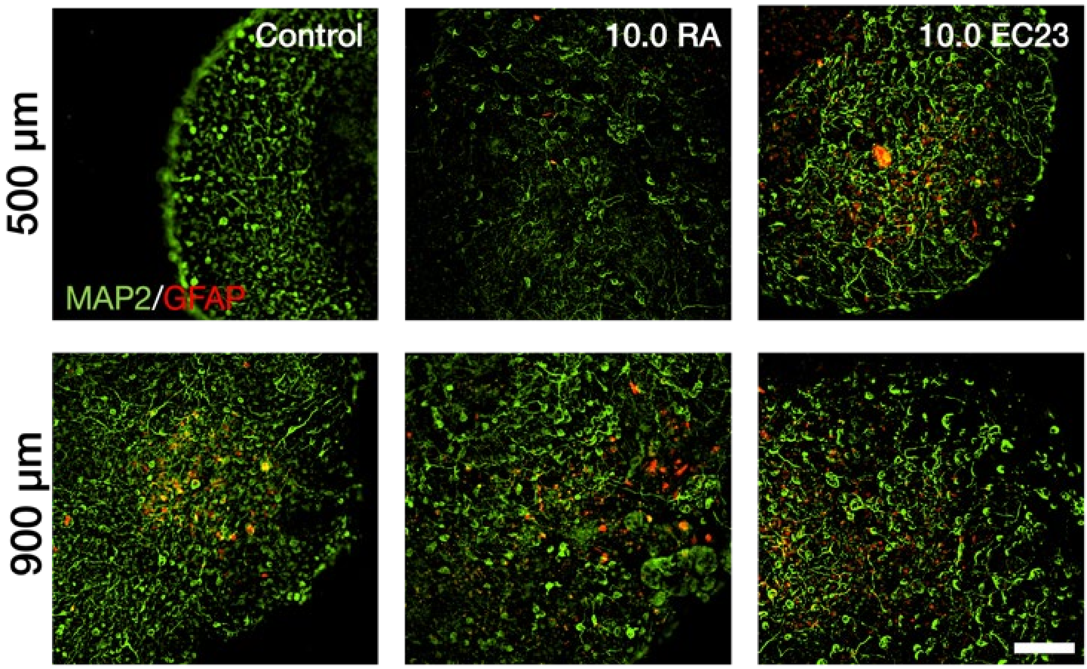

Figure S3. Immunofluorescence micrographs of neurons (MAP2, green) and astrocytes (GFAP; red) after 4-day exposure to retinoic acid (RA) or EC23 and 14 days of differentiation of small (500 µm) and large (900 µm) embryoid bodies on-chip. Scale bar, 100 µm.
